# Resilience to Demixing and Phase Segregation in Perovskite Solar Cells under Light–Dark Cycles and Temperature

**DOI:** 10.1021/acsenergylett.5c00232

**Published:** 2025-04-15

**Authors:** Alessandra Alberti, Salvatore Valastro, Elisa Nonni, Fabio Matteocci, Lucio Cinà, Aldo Di Carlo, Antonino La Magna

**Affiliations:** † CNR-IMM, Zona Industriale Strada VIII n.5, 95121 Catania, Italy; ‡ C.H.O.S.E. (Center for Hybrid and Organic Solar Energy), Electronic Engineering Department, University of Rome Tor Vergata, Via del Politecnico 1, 00118 Rome, Italy; § Cicci Research s.r.l., Via Giordani n.227, 58100 Grosseto, Italy; ∥ CNR-ISM, Area di Ricerca di Tor Vergata, via Fosso del Cavaliere n.100, 00133 Roma, Italy

## Abstract

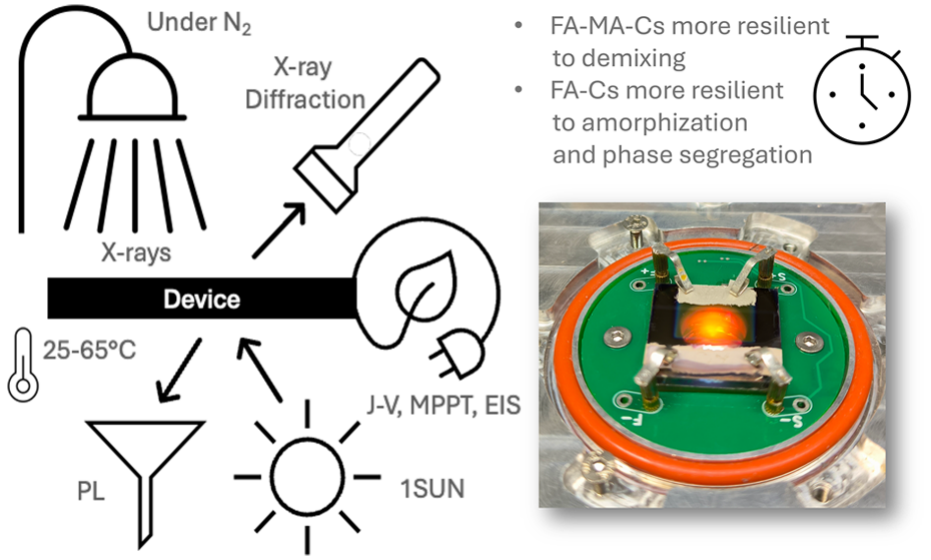

Light soaking impacts perovskite solar cells, causing cation rotation, octahedral distortion, and weakened hydrogen bonding. Using a unique *in-operando* setup for ISOS protocols, we monitor structural, optical, and electrical responses under prolonged light exposure, revealing progressive average changes without sample reloading uncertainties. Over 20 h intervals, light-induced lattice deformation causes progressive local demixing, partially reversible in dark, and residual amorphization that hinders electrical recovery. Lattice expansion and bandgap red-shift indicate increasing iodide local enrichment, while a bandgap blue-shift occurs under heating. FA-MA-Cs-perovskites resist to this ionic demixing more than FA-Cs. Sunlight is the primary trigger for that, surpassing the effects of bias or induced heating. Stress tests at 65 °C drive both formulations from demixing to irreversible phase segregation, with FA-Cs devices showing greater structural and electrical resilience than FA-MA-Cs. Since a demixing–remixing interplay governs the device operation, we recommend tracking it using *in-operando* protocols over 24–48 h of unaccelerated sunlight–dark testing.

Light soaking effects on Perovskite Solar Cells (PSC), extensively studied as reviewed by G. Zhang,^1^ have shown both beneficial and detrimental impacts. Benefits include enhanced photoluminescence, extended carrier lifetimes, and defect curing. However, light soaking can also lead to voltage and current losses, resulting in a performance decline. Some effects are reversible, as reported in ref (2), where the red-shift of the photoluminescence (PL) peak in MAPb(Br_*x*_I_1–*x*_)_3_, along with a monotonic increase in intensity under one sun constant illumination, reverts to the initial state in dark conditions. Concomitant with the red-shift, they also observe a decline in the open circuit Voltage (*V*_oc_). Reference (3) reports that bromide atomic concentrations below 50% relative to iodide (*x*_Br_ < 0.5) prevent intrinsic halide segregation, although strain may still play a role. Recent evidence of light-induced red-shift in the PL peak, accompanied by a decline in *V*_oc_, and mitigating solutions with K^+^ and Rb introduction in ref (4) on FA_0.85_MA_0.15_Pb(I_0.85_Br)_3_. Reference (5) discusses synchronized electro-optical findings in single-halide perovskites, linking *V*_OC_ losses to changes in the quasi-Fermi level splitting. In mixed-halide compositions with high Br content (e.g., FA_0.90_Cs_0.1_Pb(I_0.65_Br_0.35_)_3_), nonradiative *V*_oc_ losses are correlated to mobile ions, indicated by the lack of correlation between PL intensity and *V*_OC_ (e.g., keeping the device at *V*_oc_ decreases *V*_oc_ while PL intensity rises and red-shifts).

Starting from Hoke’s seminal paper, literature indicates that the magnitude changes and (in some cases) the derivative sign of light-soaking-affected parameters depend on perovskite formulation, material preparation methods (varying across laboratories), and stimulus conditions.^1^ They could also be time-dependent in different time scales of the experiment. For a fixed composition, perovskites respond variably to light due to intrinsic or induced mobile defects evolving during operation. Using a laser instead of simulated sunlight can alter findings, as hot carrier generation and subsequent thermalization shift excess energy to phonons, enhancing ion-phonon scattering.

## Atomic-Scale Effects of Light Soaking in Literature

Light soaking induces modifications at the atomistic scale, such as organic cation rotation, octahedra distortion^6^ and weakening of the hydrogen bonding.^7^ Less-distorted Pb–I–Pb bonds or elongated Pb–I bonds under light illumination due to electrons populating bonding states in the conduction band and holes vacating antibonding states in the valence band have been hypothesized by H. Tsai^8^ at the origin of the light-induced lattice expansion frequently observed in differently formulated perovskites, such as MAPbI_3_,^9^ CsPbBrI_2_,^10^ and FA_0.7_MA_0.25_Cs_0.05_PbI_3_.^8^ Single and double halide perovskites are thus affected. Photogenerated carriers weakening the hydrogen bonding between the amine group and the iodine ion, justified the giant photostriction (lattice expansion) observed in MAPbI_3_.^9^ Compared to MA-, Cs-, and PEA (Phenyl ethyl ammonium)-containing formulations, FAPbI_3_ seems less or unaffected by lattice expansion upon illumination, and a stronger hydrogen bonding with the ionic cage would explain this enhanced stiffness.^10^ Unlike lattice expansion induced by light soaking, FA-, MA-, Cs-, and PEA-based perovskites all exhibit lattice expansion upon heating. From the rich literature landscape,^1,11^ a general paradigm suggests that light soaking effects can vary by mixing or doping A-site cations. Additionally, both single-anion (e.g., I) and multianion (e.g., I–Br) formulations are sensitive to cumulative irradiation.

Light-induced lattice distortion then can be correlated with ionic migration and consequent demixing (e.g., in Cs_0.08_ MA_0.12_FA_0.80_ PbI_2.64_ Br_0.36_)^12^ that are light-intensity dependent and depth-dependent, with exchange/migration of ions between grains being argued; polaron-assisted local demixing under nonequilibrium conditions (e.g., in MAPb(I_*x*_Br_1–*x*_),^13^ wherein confined polarons generated by single photoexcited charge stabilize iodide-rich clusters; phase segregation by ionic migration, a process reported as irreversible^14^ or reversible;^2^ defect-assisted photoinduced halide segregation.^15^ Current knowledge stems from a combination of cross-correlated ex-situ experiments, advanced *in-situ* spatially resolved electro-optical investigations (e.g., cathodoluminescence^12^), time-resolved structural-optical (e.g., XRD-PL) analyses, PL microscopy and super-resolution optical imaging,^16^ confocal photoluminescence microscopy with chemical imaging and ToF-SIMS (secondary mass spectroscopy),^17^ nanoscale-resolved fluorescence lifetime imaging microscopy (FLIM),^18^ in some cases supported by molecular dynamics simulations. Understanding the mutual correlation among structural, optical, and electrical effects from light soaking in device operation is complex and partially incomplete. Some phenomena remain unclear, calling for new analytical methods for deeper insights. Advances in this area are scientifically and technologically significant. Additionally, multications and multianions lead halide perovskites, prioritized by photovoltaic companies,^19^ require research to predict their long-term behavior under operative conditions.

## *In-Operando* Diagnostics Accelerating Knowledge

Quite recently, *in-operando* analytical approaches have been explicitly invoked^20^ to disentangle mutually correlated effects of different natures. Their development responds to the urgent need for a better understanding of photo- and voltage-induced generation, transport, and annihilation of ionic defects under operational conditions.^21^*In-situ* and *in-operando* characterisations have been recently reviewed by R. Szostak,^22^ wherein X-ray Diffraction (XRD) and current density-voltage (*J*–*V) analyses* are combined over time to investigate phase transition and degradation issues. Electro-optical *in-operando* studies can be found in ref (5). Another survey on *in-situ* and *in-operando* methodologies is in ref (23), wherein the value of constant monitoring alterations in PSC components or complete devices has been highlighted.

A recent paper^24^ explored strain evolution in PSCs during accelerated stability tests at 2.5 V (>2*V*_oc_) under continuous light irradiation at 0.3 sun. Quasi-ISOS-V^25^ protocols have been applied using a cold white LED at 0.3 sun, through in-situ XRD, PL, and quasi-in-situ EIS. They observed a monotonic lattice expansion in Rb_0.05_Cs_0.05_(FA_0.83_MA_0.17_)_0.95_Pb(I_0.83_Br_0.17_)_3_ perovskites, accompanied by a blue-shift in PL peaks and concurrent solar cell degradation. Although the addition of organic molecules mitigated lattice expansion during stress tests, initial lattice compression was insufficient for long-term stability.^24^ The loss of XRD and PL peak intensity in the additive-engineered perovskite was associated with a crystallinity loss or decomposition.

Significant efforts have been directed toward conducting characterization under *in-operando* conditions. However, implementation to date has faced numerous limitations. Beyond methodological challenges, these constraints also affect the generality and transferability of the findings. Aligning results from laboratories for comparative evaluation and durability prediction through systematic ISOS protocols’ application on devices under unstressed and stressed conditions is urgently needed, as addressed in ref (25) .

To enable this and enhance insight accessibility beyond previous efforts, we designed a unique *in-operando* setup to analyze ready-to-use devices under realistic conditions of light soaking. All ISOS protocols can be applied under precalibrated 1 sun illumination, except for ISOS-O (outdoor stability) and ISOS-T-3 (thermal cycling −40 + 85 °C). The initial part of the study, focused on ISOS-LC protocols for light cycling, consists of unaccelerated testes, with light–dark cycles conducted for 90 h to explore light-soaking effects. In the second part, accelerated stress tests at 65 °C under 1 sunlight are applied to investigate intrinsic temperature-induced effects, as in ISOS-L2I protocols.^25^ The results varied between the two experimental conditions.

We studied light-soaking effects in typical multication multianion perovskites integrated into semitransparent devices for tandem solar cells. The observed lattice expansion has been correlated to parameters such as sunlight soaking, bias at the maximum power point, and temperature. Demixing phenomena have been observed and characterized in comparison to phase segregation. Material property restoration and the potential of electrical parameter recovery have been discussed. Based on the gained knowledge, tailored unaccelerated *in-operando* tests could be systematically applied to any perovskite formulation, fabrication protocol, and device layout to evaluate resilience toward demixing.

## Device Choice and Experiment Aims

**Figure 1:**
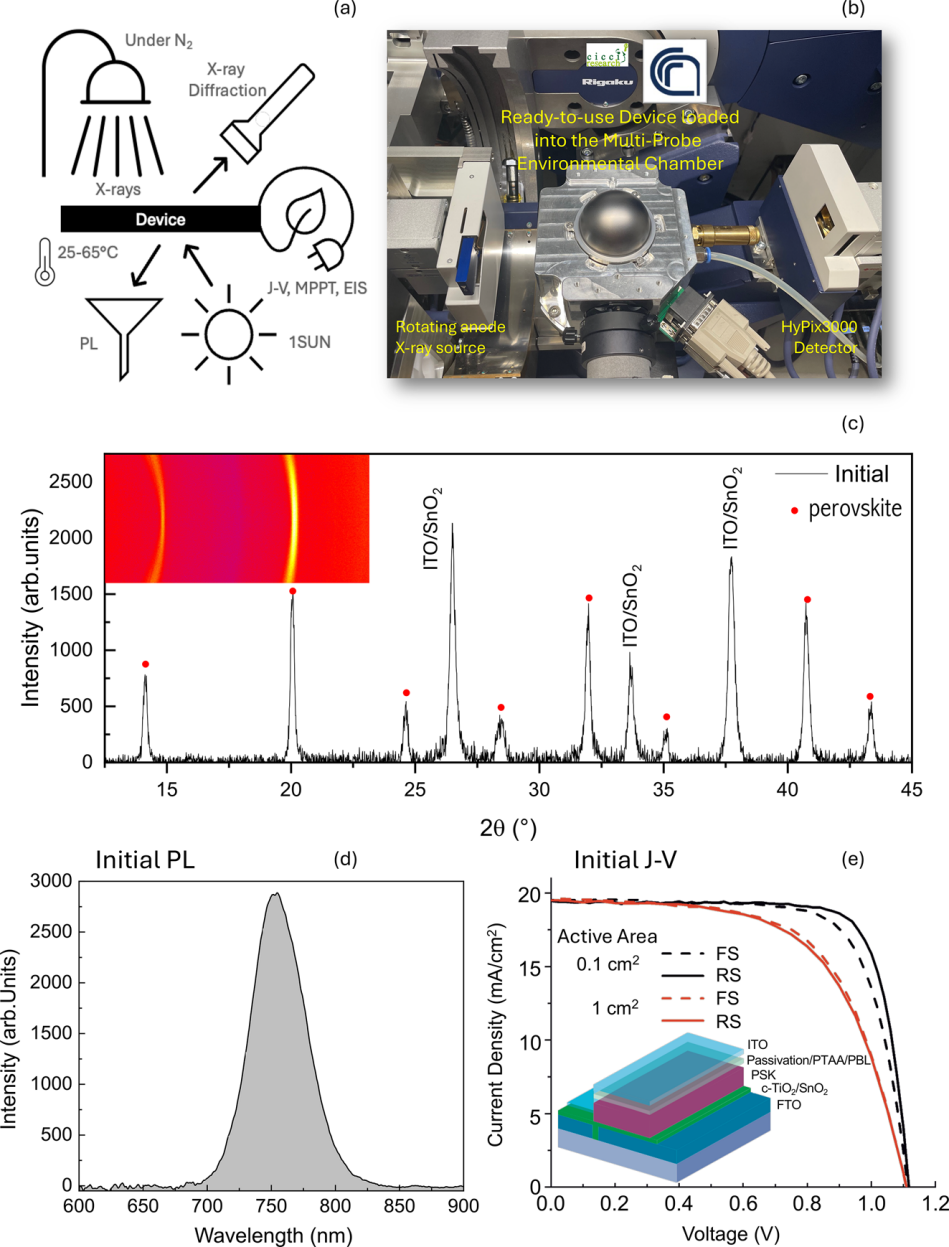
*In-operando* setup for ISOS-D, ISOS-V, ISOS-L, ISOS-T, ISOS-L2I, ISOS-LC, and ISOS-LT: schematic (a) and image (b) on combined *J*–*V*, MPPT, EIS, PL, and XRD analyses, along with temperature monitoring and setting under a controlled N_2_ atmosphere. *J*–*V* curves are acquired on complete devices with a standard layout. The Cu–Kα X-ray source with a rotating anode and parabolic mirror provides a parallel monochromatic X-ray beam at 8.05 keV with a high photon flux of 2.3 × 10^10^ photons × s^–1^ × cm^–2^, 1 order of magnitude above what is generated by standard X-ray tubes. The device is placed at a constant temperature (25 or 65 °C) through a Peltier heating–cooling system inside a graphite dome under a dynamic flux of dry N_2_ that preserves the device from extrinsic degradation. This specially designed setup enables simultaneous structural-optical-electrical analyses. (c) Initial XRD pattern of the full device (inset in e), including the top electrode, integrating a FA_0.83_Cs_0.17_Pb(I_0.83_ Br_0.17_)_3_ layer; (d) initial PL and (e) initial *J*–*V* curves in forward (FS) and reverse (RS) scan, all acquired in the setup shown in (a) and (b) and device layout in the inset.

The specialized setup used to conduct the *in-operando* experiments, detailed in the Materials and Methods section in the Supporting Information, is illustrated in Figure 1a,b. The provided comprehensive overview on structural, optical, and electrical parameters is unmatched and supported by state-of-the-art analytical facilities. The setup simultaneously allows tracking overtime high-brilliance-XRD (2.3 × 10^10^ incident photons × s^–1^ × cm^–2^ rotating-anode source and bidimensional 10^6^ cps/pixel detector), PL (with a 532 nm laser source), *J*–*V*, Maximum Power Point (MPPT), and Electrical Impedance Spectroscopy (EIS) under a calibrated solar simulator operating at 1 sun, with the device placed under controlled environmental conditions (pure dry N_2_) and temperature.

At the heart of the *in-operando* setup is the continuous data collection over a fixed device area. This eliminates the dominant statistical error from device reloading, ensuring high reliability in detecting small parameter variations and reliable cross-correlation. In Figure 1c–e, the initial structural-optical-electrical output data in a fresh device are shown.

**Figure 2:**
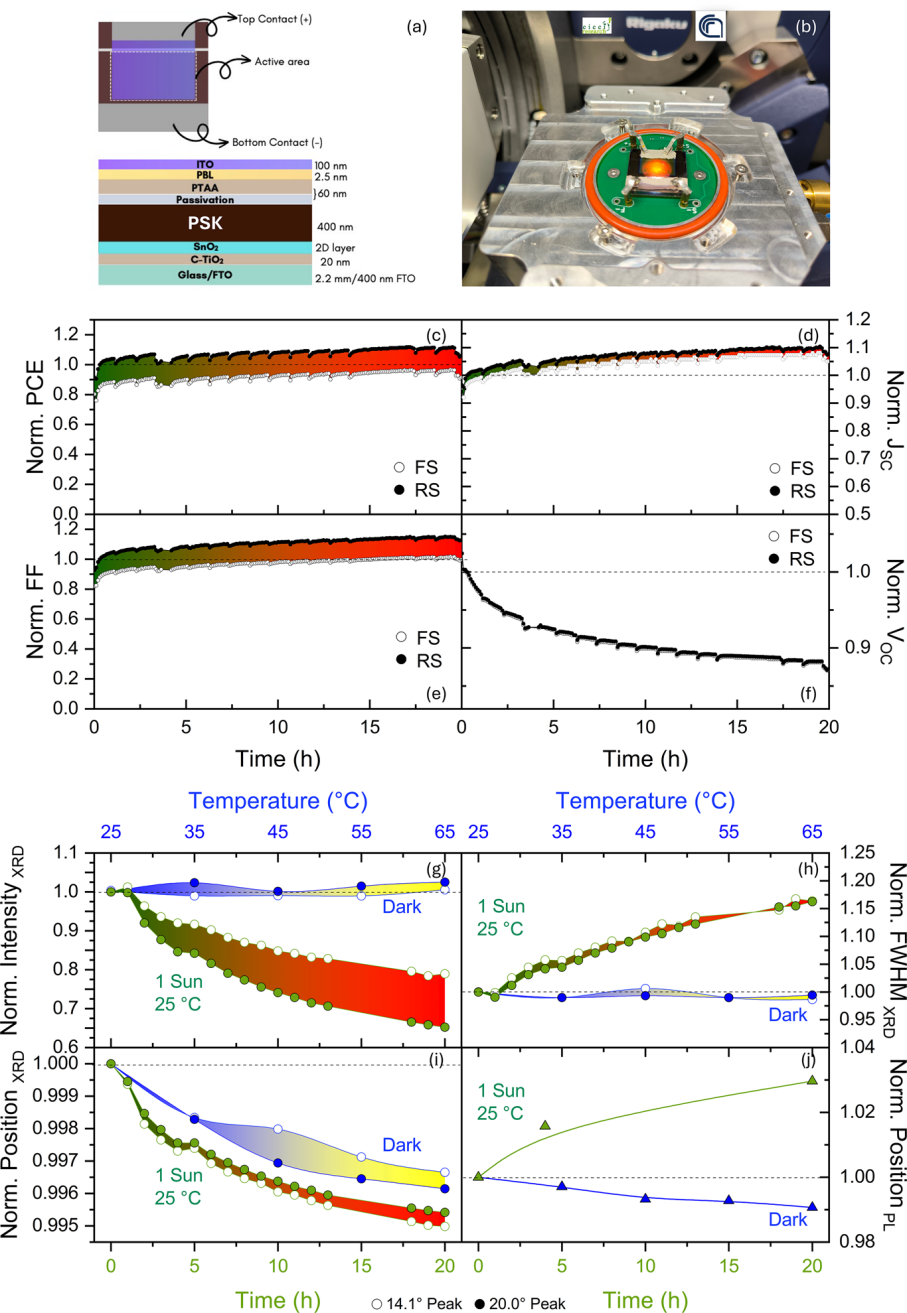
(a) Schematic of the semitransparent perovskite solar cells integrating FA_0.83_Cs_0.17_Pb(I_0.83_Br_0.17_)_3_ perovskite (PSK) and (b) picture of the real device as loaded into the environmental chamber under electrical connections and illuminated by a simulated sunlight (1 sun). (c–j) Simultaneously collected data during device operation using the operando setup in Figure 1. (c–f) Progressive *J*_sc_ rising and *V*_oc_ reducing extracted from *J*–*V* curves in forward (FS) and reverse scan (RS) along the 20 h of simulated sunlight soaking denote cumulative changes in the perovskite material (same trend from MPPT in Figure SI5). (g–j) Charts comparing data from XRD (position in degrees) and PL (position in nanometers) in the normalized scale that provide key readings to disentangle light soaking from thermal effects, the first producing a red shift of the PL peak (Δ*E*_g_ = −47 meV → 3% of the starting value) while the second causes an opposite effect (Δ*E*_g_ = 15.6 meV → 1% of the starting value). Lattice parameters are less affected by pure heating than by light soaking. See also Figure SI3.

We explored light soaking in SemiTransparent Perovskite Solar Cell devices (ST-PSC),^26^ integrating typical mixed halide perovskite formulation largely used in the literature, with composition FA_0.83_ Cs_0.17_Pb(I_0.83_Br_0.17_)_0.3_ or FA_0.78_MA_0.16_ Cs_0.06_ (PbI_0.83_ Br _0.17_)_3_. Those formulations are expected to be affected by light soaking.^23^ The device features an ITO top electrode for optical semitransparency, enabling BIPV, agrivoltaics, and perovskite/c-Si tandem (Supporting Information, Note 1). The device layout has been optimized for two-terminal mechanically stacked perovskite/c-Si tandem solar cells,^26^ as shown in Figure SI1. The ST-PSC devices showed PCE values of 17% and 13.4% for 0.1 cm^2^ and 1 cm^2^ active areas, respectively (Figure 1e). Although not preferable,^27^ full-area gold top contacts remain compatible with the used high-brilliance X-ray diagnostics, as they do not obscure the underlying material.^23^ The PSC inside the analytical system (without the closing graphite dome) is shown in Figure 2a,b.

The full experimental protocol is detailed in Table SI1. The first 20 h of the experiment are dedicated to the diagnosis of light-soaking effects under 1 sun continuous exposure, followed by light–dark alternation to investigate the extent of parameter reversibility under unaccelerated variable conditions. From minute 90, a second part of the experiment starts dedicated to accelerated stress tests at 65 °C under nitrogen conditions, as for the rest, to explore the intrinsic behavior of the material.

Figure 2c–j shows the first batch of comparative results taken from the *J*–*V* curves with a double aim. A device has been prepared and placed at a fixed temperature of 25 °C into the dome under a dry nitrogen environment. It has been biased by a four-point probe and monitored over time under 1 sunlight illumination. Simultaneously, the X-ray beam probed an area of 5 mm × 1 mm over the device from the ITO side inside the entire device structure. We aim to monitor light-soaking effects within 20 h before dark storage. The second purpose is to isolate heating effects. To do this, a fresh device is placed in the analysis chamber under dry nitrogen and monitored optically and structurally at temperatures between 25 and 65 °C in dark conditions.

## Light Soaking vs Pure Heating

During sunlight irradiation, a PCE increase piloted by the rise in *J*_sc_ and FF has been observed over time. The *V*_oc_ has a countertrend, as is often observed in literature.^2,4,5^ Parameters from MPPT (Figure SI2), denoting the device behavior at a typical working point, follow the same trend. Although some conclusions could be debated based on the literature, the synchronized structural and optical parameters recorded on the perovskite layer enable direct interpretation. Along the timeline, a leftward shift of the diffraction peaks (Figures 2i and SI3a,b) has been observed, resulting in a maximum *d*-spacing variation of +0.5% in 20 h of illumination. This phenomenon can be regarded as a lattice expansion during light soaking. It is driven by a slow kinetics in the time scale of hours. A progressive red-shift of the PL peak has been concurrently observed, as shown in Figures 2j and SI3c,d. This shift explains the *J*_sc_ increase and the *V*_oc_ decrease recorded in the device, which are all associated with a shrinkage of the bandgap.^28^ A widening of the bandgap under pure heating in conservative environments (e.g., nitrogen) is observed in literature for various perovskite formulations.^29,30^ Disentangling lattice expansion from thermal expansions during device operation^8,31^ is necessary since light and heat both impact under sunlight,^10^ and *in-operando* studies are suitable for that. To elucidate this point, it has been observed (Figures 2g–j and SI3g,h) that under pure heating, without illumination, a minor leftward shift of the XRD peaks occurs similarly to the case under illumination, whilst the PL peak is blue-shifted. Differently from light soaking, a final peak position proportional to the temperature is promptly achieved. We further notice that XRD peaks’ intensity and full width at half maximum (FWHM) remain constant during pure heating while under light soaking peak intensity, initially constant for about 1.5 h, gradually decreases. The FWHM monotonically increases, mirroring that a disorder is progressively rising into the material.

Notably, we found (Figure 2i) that the final shift of the XRD peaks after 20 h of light soaking exceeds that observed under a constant temperature of 65 °C. Excluding that the temperature of the device is progressively rising during 20 h of soaking and having verified that the final temperature is well below 65 °C, it is argued that a progressive demixing process is occurring in the material that redistributes the species, primarily by Br^–^ and I^–^ against-gradient diffusion.^32^ This counter-gradient diffusion has a possible driving force in the deformation field associated with large polaron formation,^33^ a consequence of the local photon absorption events. Indeed, joined experimental evidence and Molecular Dynamics simulations in literature have demonstrated that polaron-induced demixing causes progressive local iodide enrichment into perovskite (sub) domains.^13^ This finally reflects the progressive left shift of the diffraction peaks. Bromide short-range migration likely contributes to (sub) domain shell amorphization. Even in iodide-based perovskites,^17^ photoinduced effects have been correlated to a net migration of iodine through confocal photoluminescence microscopy and local chemical imaging. Cations can play an additional role in this diffusion interplay.^14,34^ Lattice expansion in mixed anions vs pure iodide perovskites reported in the literature will be further commented on (see also Supplementary Note 2).

Demixing does not necessarily lead to phase segregation as long as the migrated species do not stably aggregate into complementary phases.^32^ They usually produce identifying footprints into the PL signal (second peak or shoulders). Local atomic demixing is expected to be partially or totally reversible, and therefore, peak positions in XRD and PL patterns could be at least partially restored.

## Light–Dark Unaccelerated Tests Disclose Partially Reversible Effects

A measure of the progressive demixing by Br migration during light soaking, reverted during dark storage, can be found in Figure SI4 based on the XRD peak shift. Accordingly, the PL peak position has a red-shift. The parameters’ partial or total recovery in the dark corroborate that compositional restoration is feasible.

During demixing, PL intensity hugely (in double cations) or slightly (in triple cations) increases during the first 4 h of light soaking, followed by a decrease (in double cations) or a steep quenching (triple cation) during an additional 16 h under sunlight (Figure SI2). Those findings, coupled with a decreasing *V*_oc_ and increasing *J*_sc_ in the double cation formulation, compared to a decreasing *V*_oc_ and slightly decreasing *J*_sc_ in the triple cation formulation, testify in favor of a higher level of generated nonradiative defects in this last case during irradiation. Br short-range migration (toward structural sinks) activated by the sunlight can be thus argued. Halides only partially reverted into the original lattice architecture under prolonged dark storage. What is left behind is a tentative share of disordered material, likely stoichiometry-dependent.

**Figure 3:**
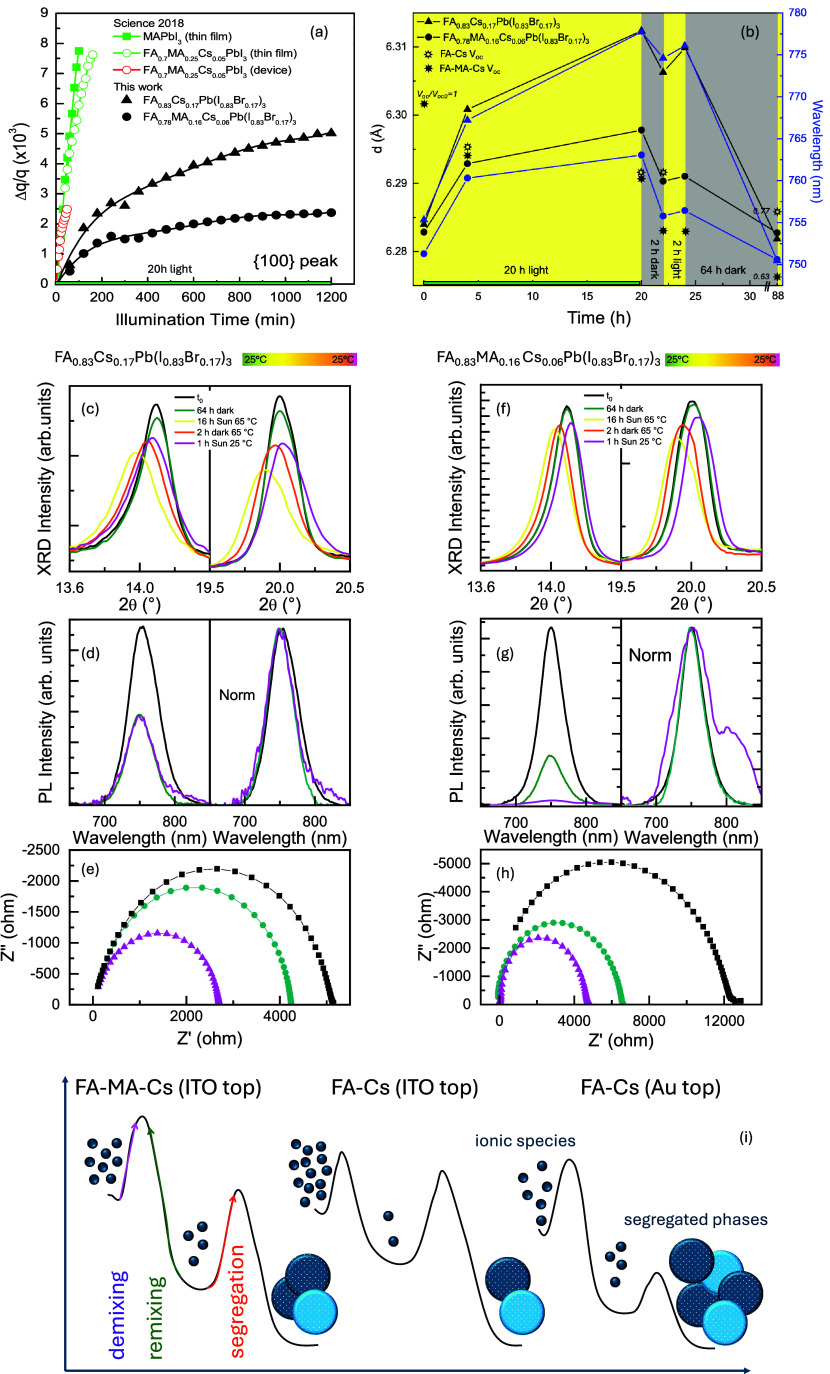
(a) Relative increase of the interplanar distance, represented by the reciprocal space vector **q**, for our perovskites compared to those reported in ref (8). All data are acquired at 25 ± 0.5 °C with a controlled temperature stage. In our work, 2θ–ω analyses are performed in dry N_2_ onto the full device placed at the maximum power point; in ref (8), GIWAXS measurements at 10^–5^ Torr vacuum yield results similar to those in complete PV devices at open circuit. Variations in identical stoichiometries stem from differing sample preparations across laboratories. Here, the probed lattice planes parallel to the sample surface remain largely unaffected by substrate constraints. Additionally, vacuum conditions may enhance the release of volatile species release. Overall, lattice expansion occurs across different environments and perovskite formulations. (b) Interplanar distance (*d*-spacing), PL peak position, and corresponding *V*_oc_ in selected region of interest for double and triple cations perovskites integrated into identical devices. *V*_oc_ losses are due to partial local demixing and amorphization. (c–h) XRD, PL, EIS data, before, during, and after the *in-operando* stress test at 65 °C under a nitrogen environment to probe thermally induced intrinsic modifications. (i) Picture of the relative interplay between ionic demixing, mixing, and segregation from the viewpoint of energetic barriers, with and without an opaque gold top electrode.

A comparative viewpoint of various formulations is shown in Figure 3a. Iodide-based thin films and devices reported in ref (8) are used in the chart. We added our data on a triple-cation formulation, fixed anions’ mixture and device layout. All formulations suffered from lattice expansion. The extent and rate of expansion are sample-dependent. Our findings comparatively indicate that triple-cation perovskites exhibit greater resilience to lattice expansion than their double-cation counterparts.

Figure 3b shows the absolute value of selected interplanar distances (*d*-spacing) to illustrate specifically the effect of the dark break. PL and *V*_oc_ data, simultaneously collected, are also shown. A dark period of 64 h is required for the initial lattice parameter and bandgap values to fully restore, a time longer than what was reported in ref (8) (30 min). Remarkably, the intensities of the XRD and PL peaks (see also Figure SI5) are partially lost and attributed to a local partial amorphization. The increased local disorder aligns with the FWHM broadening observed in 2 h. Thereby, to the extent that short-range demixed ions return to sharing the “initial” perovskite lattice, structural and optical restoration by device storage in the dark is possible (no morphological changes observed after prolonged light soaking, see Figure SI6). Although *d*-spacing and PL peak positions get back for both perovskite formulations, PL is more quenched in FA-MA-Cs than in FA-Cs perovskites. Triple-cation perovskites are also less responsive in preserving the electrical parameters (e.g., *V*_oc_, *J*_sc_, PCE).

## Temperature-Accelerated Tests Disclose Irreversible Effects

An accelerated stress test is applied at 65 °C under N_2_ to explore eventual thermally activated intrinsic transformations (Figure 3c–h). At the end of the experiment, XRD and PL have been irreversiblly modified, with PL gaining a tail (FA-Cs) or a shoulder peak (FA-MA-Cs). Those features denote the segregation of a perovskite with a lower bandgap than the original one. According to this change, the XRD peaks are shifted and deformed by an additional convoluted contribution. In both cases, the electrical parameters decline (Figure SI2) and, accordingly, the Nyquist plots from EIS analysis (1 Hz to 10 kHz at open circuit) show a reduction of the shunt resistance (Real part of Z impedance), generally associated with recombination phenomena in the active layer. Overall, the accelerated test induced irreversible phase segregation. The final PCE drops to 20% of the initial value in FA-MA-Cs devices and 40% of that in FA-Cs devices. FA-Cs devices with full-area gold top contacts (no fingers) replacing ITO show an overall constant decline of performances (Figure SI7), beginning at room temperature during light soaking and further amplified under thermal stress, likely driven by Au diffusion-related effects.^27^ An overall picture on mixing, demixing, and phase segregation is provided in Figure 3i.

## Temperature, Sunlight, and Bias in Short

A targeted experiment was conducted on a fresh sample at 65 °C to isolate the effects of temperature, bias, and light (Figure SI8). A peak shift was observed after 1 h of illumination without bias. Following an additional 1 h under sunlight and MPPT, no significant changes occurred. Another hour without illumination and bias restored the peak, while a subsequent hour under bias at the MPP left the peak position unchanged. Overall, the findings unequivocally disclose that sunlight, more than bias and beyond heating, causes lattice modifications.

## Conclusive Remarks

In summary, we collected synchronized structural-optical-electrical data on ready-to-use PSCs via an advanced *in-operando* multi-probe setup to capture cumulative time-dependent changes under operative sunlight exposure. It supports ISOS-D protocols for dark storage recovery, as well as ISOS-L2I for temperature effects, and ISOS-LC with ISOS-L for light cycling and soaking, enabling comprehensive device analysis.

Typical double- and triple-cation perovskites with iodide- bromide formulations were selected for integration into semitransparent devices, ensuring compatibility with tandem solar cells. The study started with light–dark unaccelerated cycles to assess light-soaking effects under realistic conditions. In the second part of the experiment, accelerated tests at 65 °C simulated extreme heating, as in the ISOS-L2I protocols. Results varied between the two conditions, highlighting the need to explore both.

The applied analytical multi-probe approach helps distinguish between pure lattice expansion (inducing a PL blue shift) and ionic demixing with iodine enrichment (leading to a PL red-shift associated with perovskite bandgap shrinkage). We demonstrate that sunlight, more than bias or heating, primarily causes demixing-related lattice expansion.

Given that demixing can impact any perovskite formulation, we conclude from literature comparisons that photoinduced halide redistribution via halide migration is a key phenomenon in both double-halide and single-halide perovskites. Furthermore, our study reveals that both double- and triple-cation formulations are susceptible to demixing, though with distinct differences in behavior. In both cases, to the extent that locally demixed ions return to sharing the “initial” lattice via ionic remixing, (partial) structural and optical restoration by dark storage is possible. Along this demixing–remixing dynamics, part of the initial perovskite volume is statistically lost, with fingerprints being PL peak intensity quenched, the XRD peak area reduced, and the shunt resistance reduced. Our findings indicate that triple-cation perovskites exhibit greater demixing resilience than the double-cation counterparts. However, both triple-cation and double-cation-perovskites experience losses in XRD and PL peak intensity as well as in open-circuit voltage (*V*_oc_). Triple-cation perovskites, in particular, were featured by a faster PL quenching and were less responsive in preserving electrical performances. To pass the threshold from demixing to irreversible phase segregation, thermal stress at 65 °C was effective in both formulations. In terms of PCE degradation, double-cation perovskite devices demonstrate higher resilience under thermal stress conditions compared to triple-cation perovskite-based devices.

In brief, perovskite behavior under operative conditions is governed by an interplay between demixing, remixing, and irreversible phase segregation, which are light intensity and temperature dependent. This ultimately makes the explored FA-Cs perovskite more prone to demixing but also to remixing at room temperature while being less susceptible to thermal phase segregation.

We emphasize the need for standardized unaccelerated tests for each perovskite formulation, fabrication protocol, and device layout to assess demixing’s impact on durability under real-world conditions. Fully *in-operando* tests offer sensitive data within 24–48 h, providing robust diagnostics and data correlation. These tests can aid in developing predictive models for the structural and optical evolution of perovskites under operational conditions, guiding mitigation strategies for extended durability.

## Data Availability

All data are available in the main text or the Supporting Information.

## Supporting Information Available

Experimental section; *In-operando* stability testing protocol with environmental conditions, temperature, illumination, and measurements over time; Schematic, *J*–*V* curve, and SEM cross-section of tandem perovskite solar cell; *In-operando* photovoltaic parameters, XRD, and PL data for FA_0.83_Cs_0.17_Pb(I_0.83_Br_0.17_)_3_ and FA_0.78_MA_0.16_Cs_0.06_(PbI_0.83_Br_0.17_)_3_ devices over time; XRD and PL data under illumination over time and under controlled heating; Lattice parameter–Br content correlation from XRD and PL shifts; Photovoltaic parameters, XRD peak shifts, and PL spectra after dark–light cycling. FIB-SEM images of as-prepared and 1 sun illuminated FA-Cs perovskite devices; Opaque perovskite solar cell schematic, stability comparison, and phase segregation illustration; XRD peak shifts under illumination and bias at MPP at 65 °C. Discussion on lattice expansion in mixed anions vs pure iodide perovskite (PDF)
