# Supplementary material for: Resilience to Demixing and Phase Segregation in Perovskite Solar Cells under Light–Dark Cycles and Temperature
Source: ACS Energy Lett. 2025 Apr 15;10(5):2259–67. doi: 10.1021/acsenergylett.5c00232 (PMC12070746; doi:10.1021/acsenergylett.5c00232)
Supplement: Supplementary file 1 — nz5c00232_si_001.pdf [file nz5c00232_si_001.pdf]

SUPPORTING INFORMATION

Resilience to demixing and phase segregation in perovskite solar cells under light-dark cycles and temperature

Alessandra Alberti[1], Salvatore Valastro[1], Elisa Nonni[2], Fabio Matteocci[2], Lucio Cinà[3]*
Aldo Di Carlo[2,4], Antonino La Magna[1]*

[1]*CNR-IMM, Zona Industriale Strada VIII n.5, 95121 Catania, Italy*
[2]*C.H.O.S.E. (Center for Hybrid and Organic Solar Energy), Electronic Engineering Department, University of Rome Tor Vergata, Via del Politecnico 1, 00118, Rome, Italy*
[3] *Cicci Research s.r.l., Via Giordani n.227, 58100 Grosseto, Italy*
[4]*CNR-ISM, Area di Ricerca di Tor Vergata, via Fosso del Cavaliere n.100, 00133 Roma, Italy*

**Corresponding author. Email*: *salvatore.valastro@cnr.it*
**Corresponding author. Email*: *aldo.dicarlo@artov.ism.cnr.it*

Materials and Methods

1. *In-operando* setup

The setup has been developed at the CNR-IMM in collaboration with Rigaku and Cicci research. It consists of a modified Smartlab equipment for high-angular-resolution X-ray diffraction that operates with a rotating 8.05 keV Cu-source, generated at 45 kV of bias and 200 mA of e-current (9 kW tube). The monochromatic beam has a high brilliance of 2.2×10^{10} photons$\times$s$^{-1}\times$cm^{-2}. The primary optics consists of a parabolic mirror for Cu-kβ stripping, soller slits and 1 mm aperture; the secondary optics has soller slits and 1 mm aperture placed before the HyPix-3000 detector (pixel size 100 µm²) operating in 1D configuration.
A multipurpose chamber (ARKEO - Cicci Research s.r.l.) has been integrated at the multi-goniometer center of the Smartlab diffractometer. It is s equipped with: a 4-wires and 4-quadrant probing system at V=-/+60V, I=+/-3A, resolution 100nV / 100 fA, speed up to 1.8Msampling/s; a multiLED solar simulator with 12 independent sources specifically made of Cool White (>4750K), Warm White (<3750K), Far UV (365nm), UV (385nm), Deep Blue (450nm), Green (510nm), Amber(590nm), Red (625nm), Hyper Red (660nm), Far Red (730nm), IR (850nm), Far IR (940nm) which illuminate the sample on an active area of 0.45 cm^2 .
The device temperature can be controlled with +/- 0.1°C of resolution in the range 10-90°C with a Peltier module having a hole in the center. The hole enables optical excitation with the

multi-LED array (calibrated sun simulator) and photoluminescence monitoring (PL). PL measurements are performed with a 520 nm free space laser source and a fibre-based spectrometer (350-1100 nm). The irradiance spectrum of the multi-LED source is calibrated at AM 1.5G. The hereafter reported figure shows the integrated power for each spectral band related to the ASTM E927-19 method.

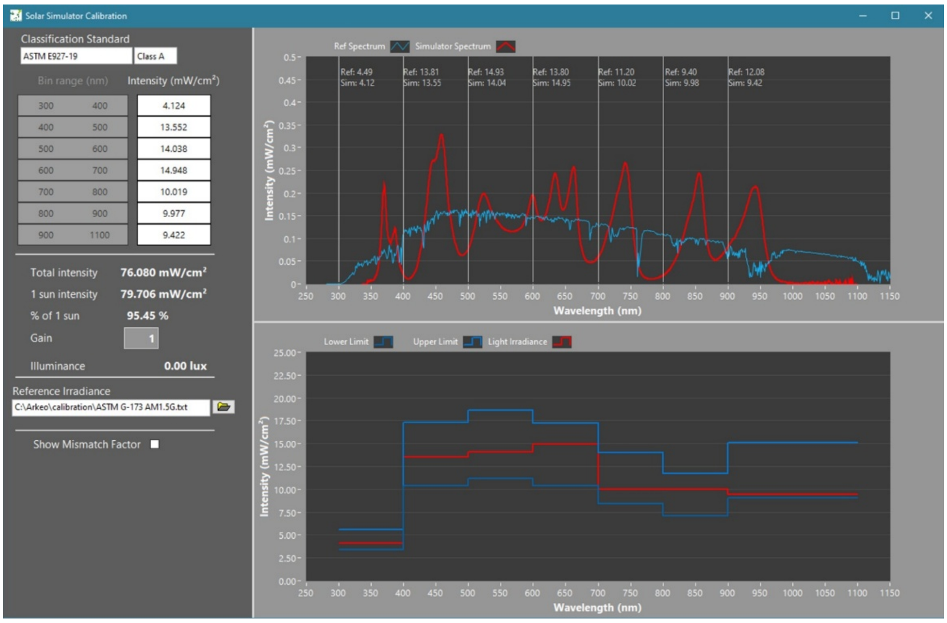

A graphite dome is used to house the device under a controlled dry N_2 atmosphere.

2. Device preparation

Semi-transparent PSC have been fabricated starting from Glass/FTO substrates (2.2 mm-thick TEC15 Pilkington, 15 Ω/square, 2.5x2.5 cm^2 substrate area). Laser etching was performed using nano-second pulsed laser (Nd:YVO_4, λ = 355 nm, 15 ns, pulsed at 80 kHz with a fluence per pulse of 715 mJ cm^{-2}) to obtain the substrate patterning for deposition of 1 cm^2 active area devices. The patterned substrates were cleaned in an ultrasonic bath, using de-ionized water/soap, de-ionized water and finally 2-propanol (10 min for each cleaning step). Screen-printed Silver contacts (DuPont 7713) are deposited over FTO photoanodes.
The 20 nm-thick TiO_2 compact layer (c-TiO_2) is deposited by spray pyrolysis at 460°C starting from a precursor solution made up of titanium diisopropoxide bis(acetylacetonate), acetylacetone and ethanol in a 3:2:45 volume ratio. SnO_2 nanoparticle-based ink at 1:10 v/v in deionized water is deposited on TiO_2 coated substrate using spin coating at 4000 rpm for 20 s. Then, the substrates are annealed at 120°C for 20 minutes. After the annealing, the substrates are transferred to a nitrogen filled glove box for the perovskite deposition.

The $FA_{0.83}\,Cs_{0.17}\,Pb\,(I_{0.83}\,Br_{0.17})_3$ double cation perovskite composition was obtained by mixing the following powder precursor amounts: 402 mg PbI_2, 134 mg $PbBr_2$, 52.96 mg CsI, 171.31 mg FAI. For triple cation $FA_{0.78}MA_{0.16}\,Cs_{0.06}\,(PbI_{0.83}\,Br_{0.17})_3$ perovskite composition: 442 mg PbI_2, 88 mg $PbBr_2$, 16.41 mg CsI, 134 mg FAI, 21.85 mg MABr. Both perovskite formulations were obtained by dissolving the precursors in 1ml of DMF/DMSO (4:1 w/w) solvent mixture.

The perovskite depositions were made by spin-coating using the solvent quenching method at 5000rpm for 35s. 150ul of chlorobenzene anti-solvent was dripped on the substrate 15s before the end of the program.
Iso-Pentylammonium chloride solution is prepared at 1mg/ml concentration in 2-propanol and deposited by spin coating at 4000 rpm for 20 s. The doped PTAA solution was prepared by dissolving 10 mg of the PTAA powder in 1 ml of toluene adding TBP (10 µl/ml) and Li-TFSI (5 µl/ml, stock solution: 170 mg/ml in acetonitrile) as dopants. The PTAA films are deposited by spin coating at 4000 rpm for 20 seconds. Then, the samples are transferred to a thermal evaporator for the deposition of V_2O_5 (2.5 nm) used as a protective buffer layer (PBL).
Finally, low-temperature ITO deposition was performed by using an industrial in-line magnetron sputtering (KENOSISTEC S.R.L., KS 400 In-Line) at $1.1 \cdot 10^{-3}$ mBar and 90W RF power. Inert Ar gas is purged in the chamber (40 sccm) during the ITO deposition to activate the Ar+ plasma. The sample holder is moved below the ITO cathode with 120 cm/min speed for 150 cycles to achieve 100 nm thickness. The active area of the cells was 1 cm^2 obtained by applying a deposition mask for the ITO electrode. A schematic of the manufacturing process is reported in Figure S where the film thicknesses and the fabrication steps are resumed.

Supplementary tables and figures

Table SI1: in-operando protocol according to ISOS Khenkin et al. Nat Energy 5, 35, 2020. In particular, we applied: Dark storage (ISOS-D), Bias stability (ISOS-V), Light-soaking (ISOS-L), Thermal cycling (ISOS-L2I), Light cycling (ISOS-LC), Solar-thermal cycling (ISOS-LT). The first 24 hours of the experiment are dedicated to the diagnosis of light-soaking effects. They are followed by 64 hours under dark conditions with the device unbiased to probe the extent of parameter reversibility. The last part of the protocol is dedicated to the stress test at 65°C.

time (h)	environment	t (°c)	illumination	MPPT	JV	XRD	PL (532nm)
0	N_2	25	dark	/	dark	y	y
0-4	N_2	25	1sun	y	Δt=5min	Δt=60min	/
4	N_2	25	dark	/	dark	y	y
4-20	N_2	25	1sun	y	Δt=5min	Δt=60min	/
20	N_2	25	dark	/	dark	y	y
20-22	N_2	25	dark	/	/	Δt=60min	/
22	N_2	25	dark	/	/	y	y
22—24	N_2	25	1sun	y	Δt=5min	Δt=60min	/
24	N_2	25	dark	/	/	y	y
24-88	N_2	25	dark	/	/	/	/
88	N_2	25	dark	/	dark	y	y
88-90	N_2	25	1sun	y	Δt=5min	Δt=60min	/
90	N_2	25	1sun	y	y	y	/
90-110	N_2	65	1sun	y	Δt=5min	Δt=60min	/
110-112	N_2	65	dark	/	/	Δt=60min	/
112-113	N_2	25	1sun	y	Δt=5min	y	/
113	N_2	25	dark	/	dark	y	y

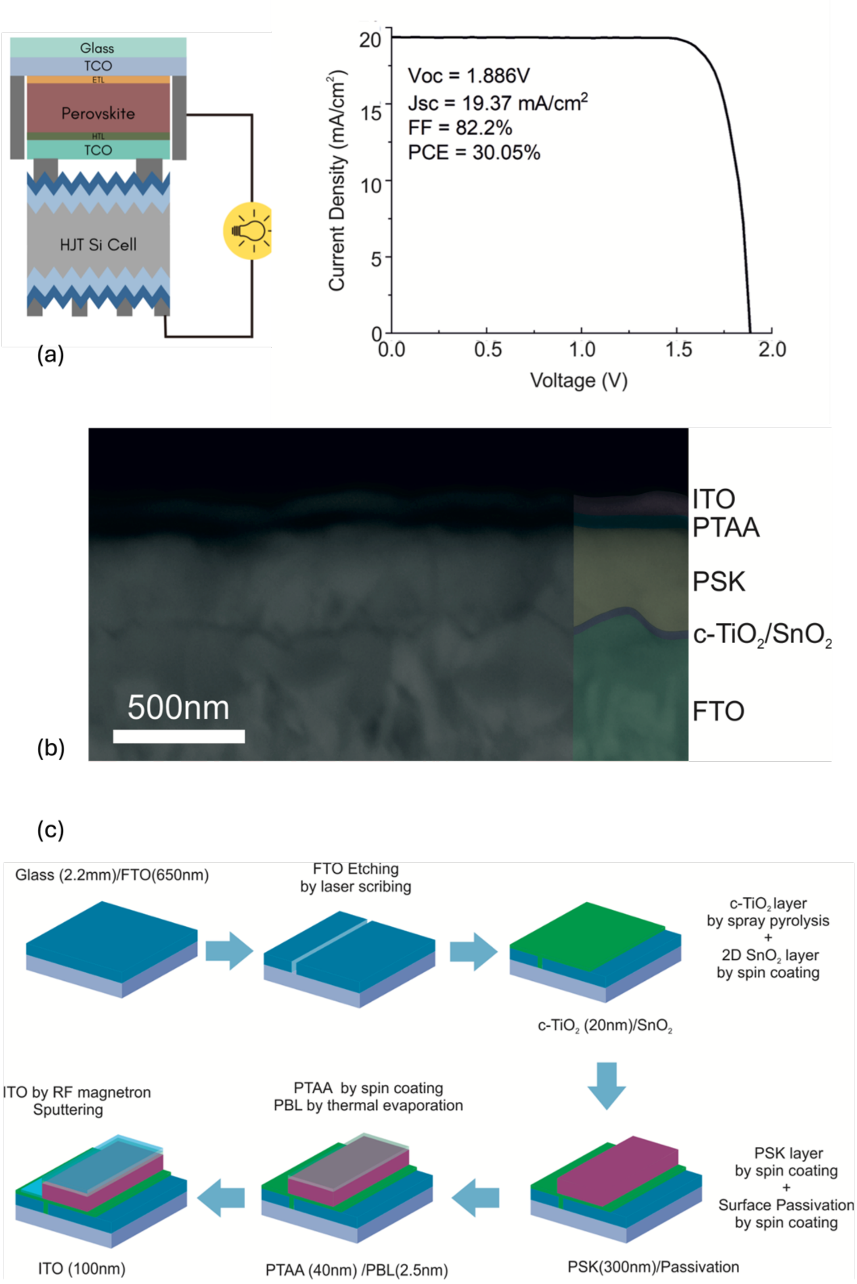

Figure SI1: (a) Schematic and J-V curve of a mechanically stacked tandem device integrating the semi-transparent Perovskite Solar Cell-ST-PSC (Lamanna et al.1) (b) SEM cross-section of the semi-transparent Perovskite Solar Cell. (c) Manufacturing Flow of an ST-PSC cell showing the stacked materials, the layers' thickness and the deposition methods adopted in the device fabrication

Supplementary Note 1

The device layout has been designed with a top electrode of ITO for optical semi-transparency to be exploited in applications such as building integrated photovoltaics (BIPV), Agrivoltaics and Perovskite/c-Si tandems. The eventual presence of additional full-area gold top-contacts is compatible with the X-ray diagnostic at high brilliance offered by the setup (differently from standard sources) since, in this case, gold does not obscure the material underneath.
The device layout has been optimised for two-terminal mechanically stacked Perovskite/c-Si tandem Solar Cells[1,] as shown in Figure SI1. The semi-transparent single-junction devices showed PCE values of 17% and 13.4% for 0.1 cm^2 and 1cm^2 active areas, respectively. In 1 cm^2 sized device, the PCE is affected by the reduced FF by the influence of sputtered-ITO back-contact on series resistance (Figure 1e). However, we demonstrate that the silver grids of the c-Si cell reduce the series resistance in the tandem cell, reaching PCE of 26.3% for $FA_{0.78}MA_{0.16}$ $Cs_{0.06}$ $(PbI_{0.83} Br_{0.17})_3$ composition[1.] Figure SI1 shows the J-V characteristic of the two-terminal mechanically stacked Perovskite/c-Si tandem using $FA_{0.83}$ $Cs_{0.17}$ Pb $(I_{0.83}$ $Br_{0.17})_3$ composition. Furthermore, the semi-transparent device stack is preferred to avoid electrode migration under prolonged light exposure using metallic top electrodes[2,3].

$FA_{0.78}MA_{0.16}Cs_{0.06}(PbI_{0.83}Br_{0.17})_3$

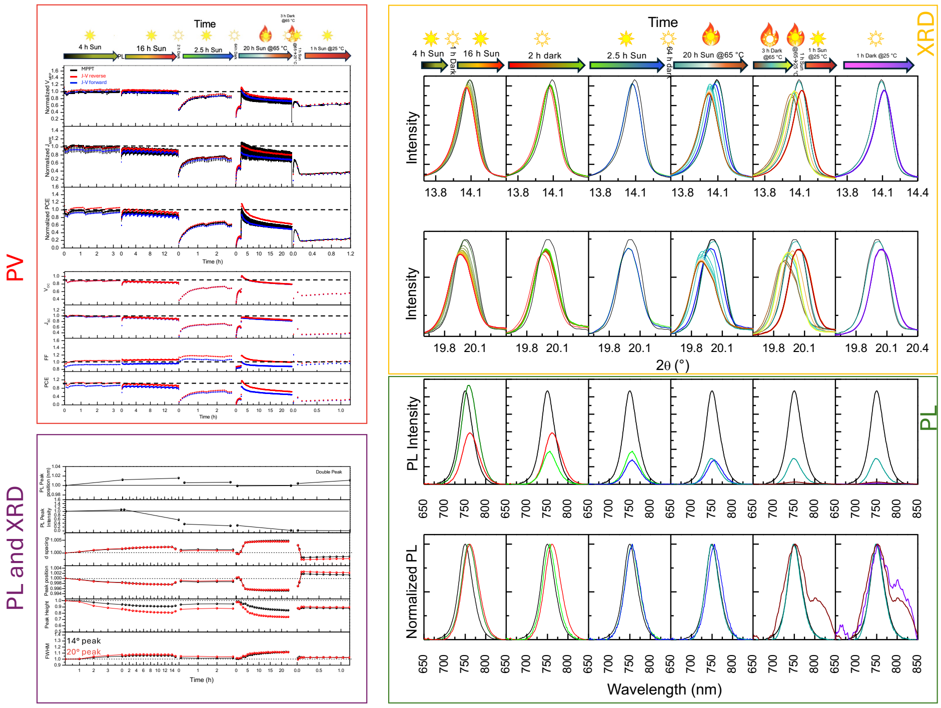

$FA_{0.83}Cs_{0.17}Pb(I_{0.83}Br_{0.17})_3$

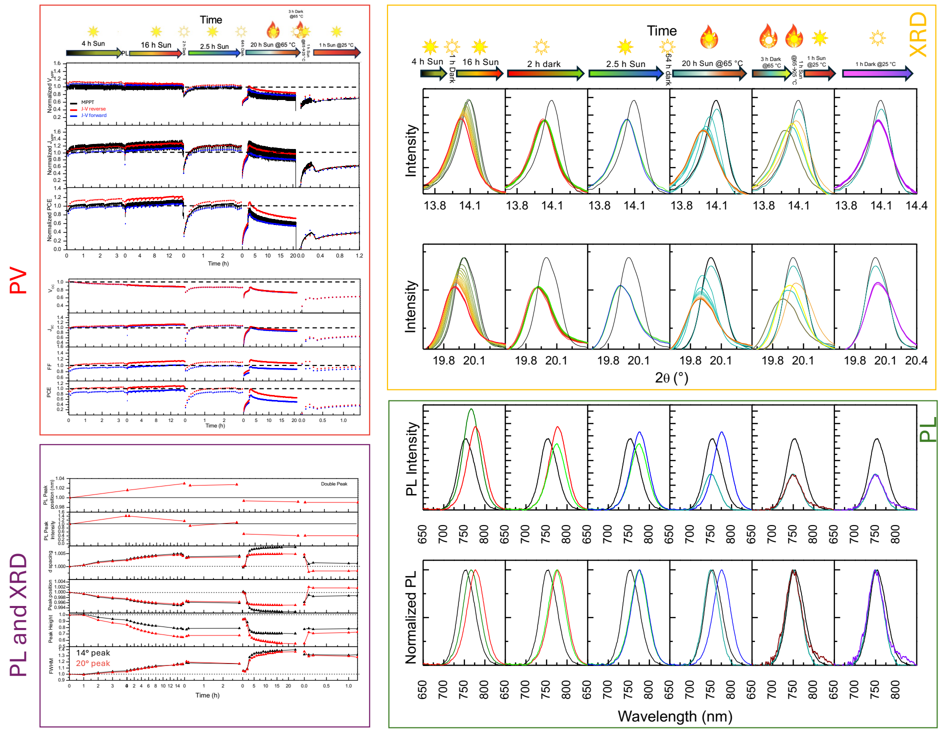

Figure SI2 In-operando experiment output from $FA_{0.78}MA_{0.16}$ $Cs_{0.06}$ $(PbI_{0.83}$ $Br_{0.17})_3$ -based devices over time, in the same experiment conducted for the *$FA_{0.83}$ $Cs_{0.17}$ Pb $(I_{0.83}$ $Br_{0.17})_3$ formulation (figure 1): Photovoltaic Parameters (V_{OC}, J_{SC}, FF and PCE), XRD and PL peaks (normalised and non)*

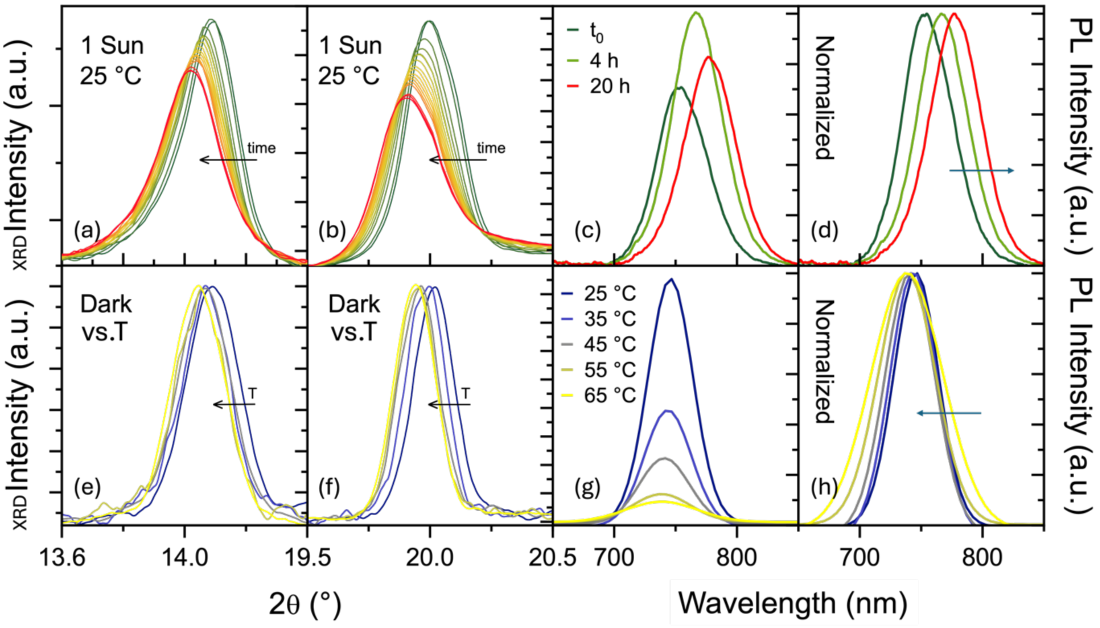

Figure SI3: XRD (a-b) and PL (c-d) data collected under 1 sun at 25°C depict an overall change of the local composition in the whole layer along 20h of analyses under simulated sunlight. Local demixing with ionic redistribution causes iodide enrichment that reflects into a shrinkage of the bandgap (and increase in the d-spacing) with indeed effects on J_{sc} and V_{oc}. XRD (e-f) and PL (g-h) measurements conducted under dark conditions and controlled heating up to 65°C reveal a shift in interplanar distance with the same sign but reduced magnitude compared to sunlight exposure, while the bandgap exhibits an opposing trend

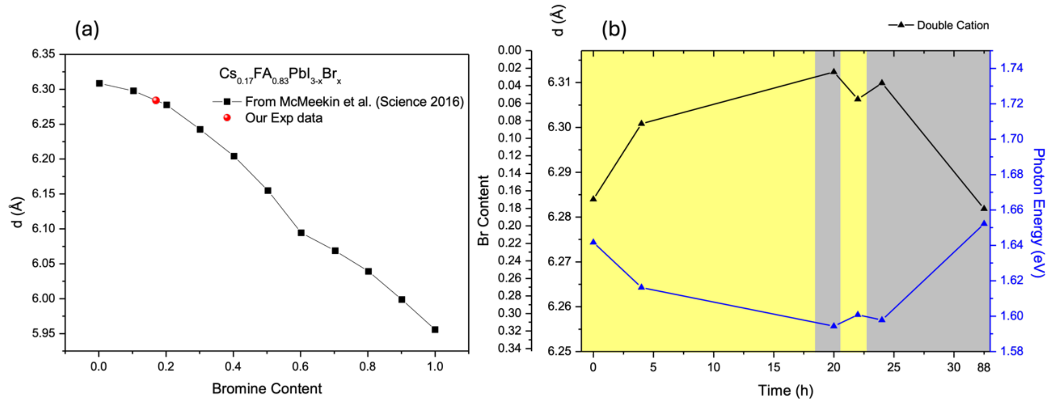

Figure SI4 (a) reference curve and experimental finding on the match between the lattice parameter and the Br content into a double-cation perovskite formulation. (b) Br content migration during light soaking (yellow regions) and dark storage (grey regions) based on the XRD peak shift. Accordingly, the PL peak position shifts and the final value corroborates the final compositional restoring.

As a note, the sign of the XRD peak shift is important to consider since a leftward shift mirrors a (reversible or irreversible) enrichment of iodide phases whilst a rightward shift would be symptomatic of bromide enrichment. The achievement can vary from composition to composition and from lab to lab.

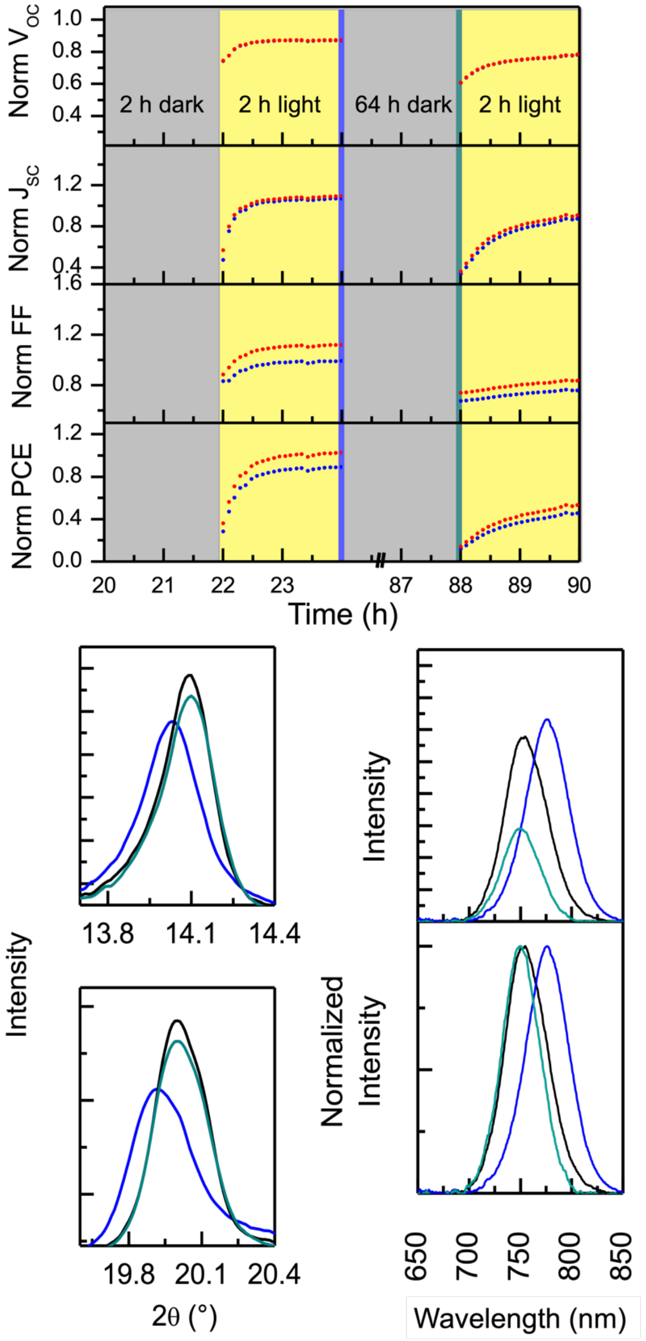

Figure SI5 Photovoltaic Parameters (V_{OC}, J_{SC}, FF and PCE, red (RS) and blue (FS)) after 2 cycles of dark: light 2:2 and 64:2. XRD peaks (14.1° and 20° peaks) and PL at t_0 (black curve), t=24 h (blue curve) and t=88 h (green curve)

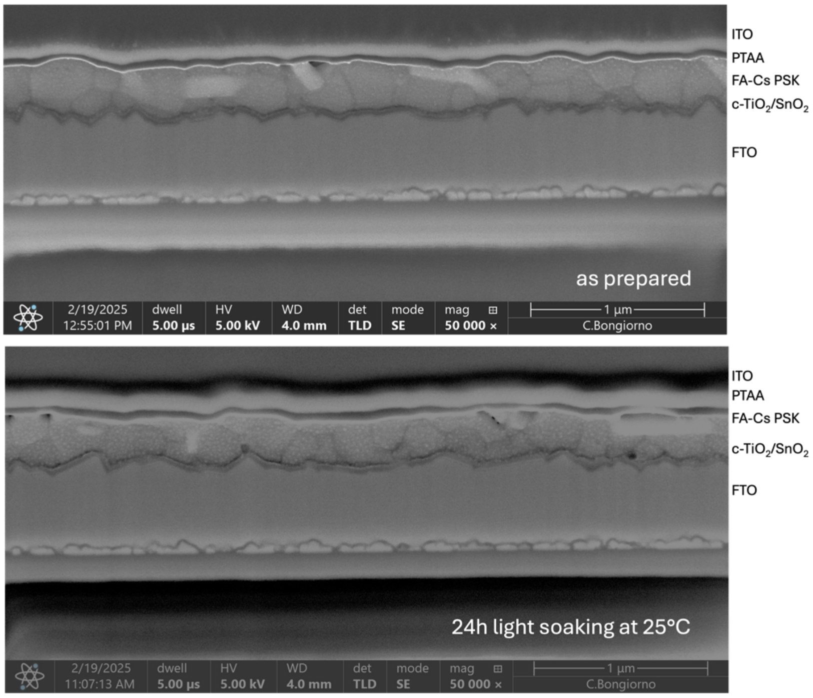

Figure SI6 FIB-SEM cross-sectional images taken on the as prepared and on light-soaked devices based on FA-Cs perovskites. FIB cross sections (Thermofisher Helios 5 UC+ system) were obtained using 30keV Ga+ ions with a sequence of a regular cross-section at 2.5nA and a cleaning cross-section at 0.43nA, after the deposition of 100nm of electro-deposited and 1.5μm of ion-deposited protective carbon layer. SEM images are acquired at 5keV and 0.2nA of electron current in secondary electron configuration using a trough-lens detector. The grains are mostly columnar, and the layers are uniform (in both pictures, small round features into the large grains come from the perovskite interaction with the e-beam). No significant morphological changes have been detected.

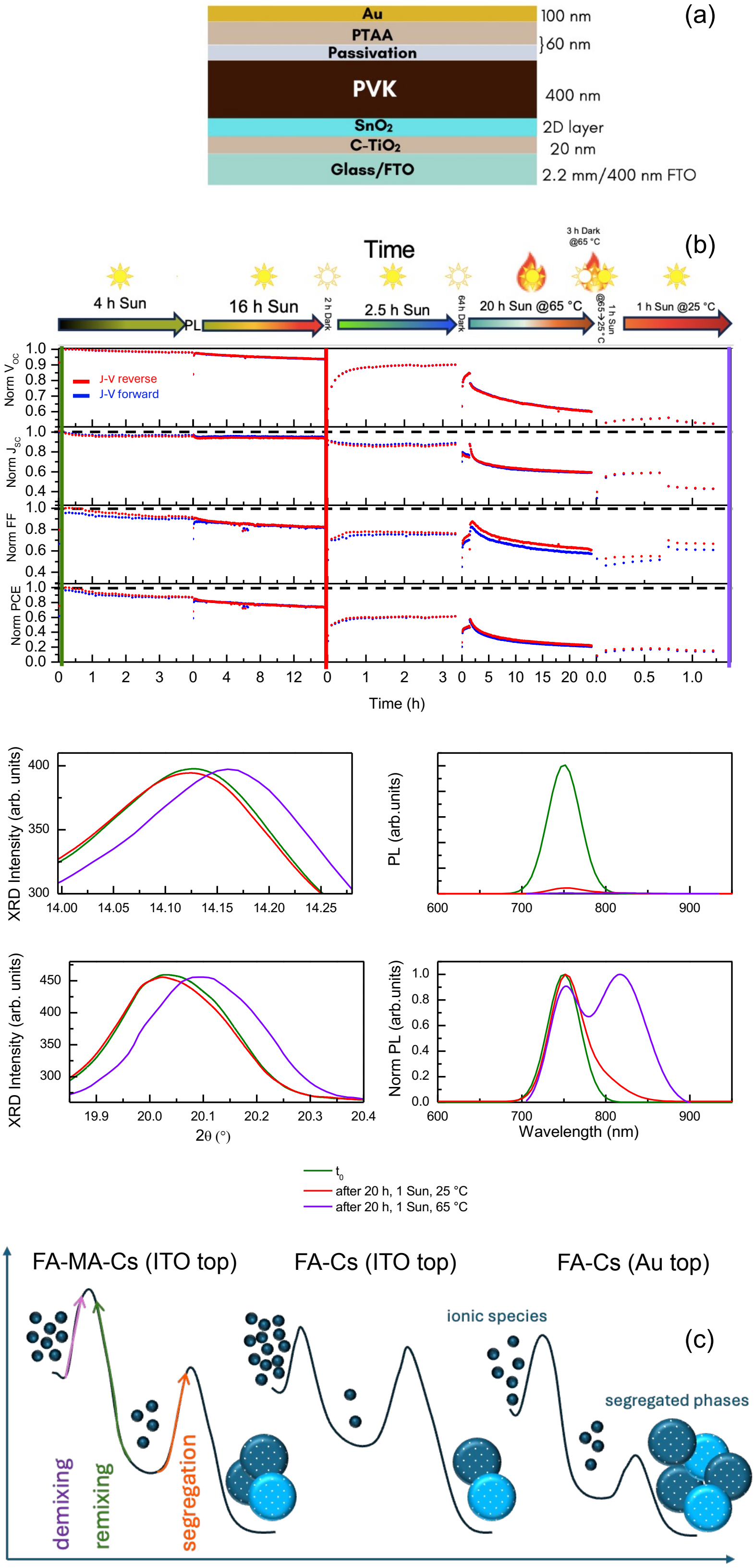

Au
PTAA
Passivation
PVK
SnO2
C-TiO2
Glass/FTO
100 nm
}60 nm
400 nm
2D layer
20 nm
2.2 mm/400 nm FTO
(a)
Time
(b)
4 h Sun
PL
16 h Sun
2 h Dark
2.5 h Sun
64 h Dark
20 h Sun @65 °C
3 h Dark @65 °C
1 h Sun @65→25 °C
1 h Sun @25 °C
J-V reverse
J-V forward
Norm Voc
Norm Jsc
Norm FF
Norm PCE
Time (h)
XRD Intensity (arb. units)
PL (arb.units)
Norm PL (arb.units)
2θ (°)
Wavelength (nm)
t0
after 20 h, 1 Sun, 25 °C
after 20 h, 1 Sun, 65 °C
FA-MA-Cs (ITO top)
FA-Cs (ITO top)
FA-Cs (Au top)
ionic species
segregated phases
(c)
demixing
remixing
segregation

Figure SI7: (a) schematic of the opaque perovskite solar cells integrating $FA_{0.83}Cs_{0.17}Pb(I_{0.83}Br_{0.17})_3$ *perovskite (PSK) and gold as top electrode. (b) In-operando experiment output: Photovoltaic Parameters (*V_{OC}*,* J_{SC}*, FF and PCE), XRD and PL peaks (normalised and non) over the time. Our results indicate that the opaque architecture is less stable than the semitransparent one. After 20 hours under 1 Sun illumination at 25 °C, all photovoltaic parameters (*V_{oc}*,* J_{sc}*, and FF) decrease, accompanied by significant PL peak quenching. Additionally, a tail appears in the PL spectrum, suggesting that phase segregation occurs at 25 °C under illumination in this architecture. In contrast, in the semitransparent design, it was only observed after thermal stress testing at 65 °C. It is well established in the literature that prolonged illumination can lead to gold diffusion into the perovskite layer, causing degradation through chemical reactions with perovskite components, such as iodine* [,4]*. In our case, gold diffusion into the perovskite may create nucleation sites for phase-segregated perovskite, triggering irreversible phase segregation even without the need for high temperatures. The thermal stress test (65 °C) causes a further drop in the PV parameters and in the PL peak intensity with the appearance of a shoulder peak, meaning the worsening of perovskite phase segregation. (c) a picture of the interplay between ionic demixing, mixing and segregation from the viewpoint of associated energetic thresholds in the cases with and without opaque gold top electrode*

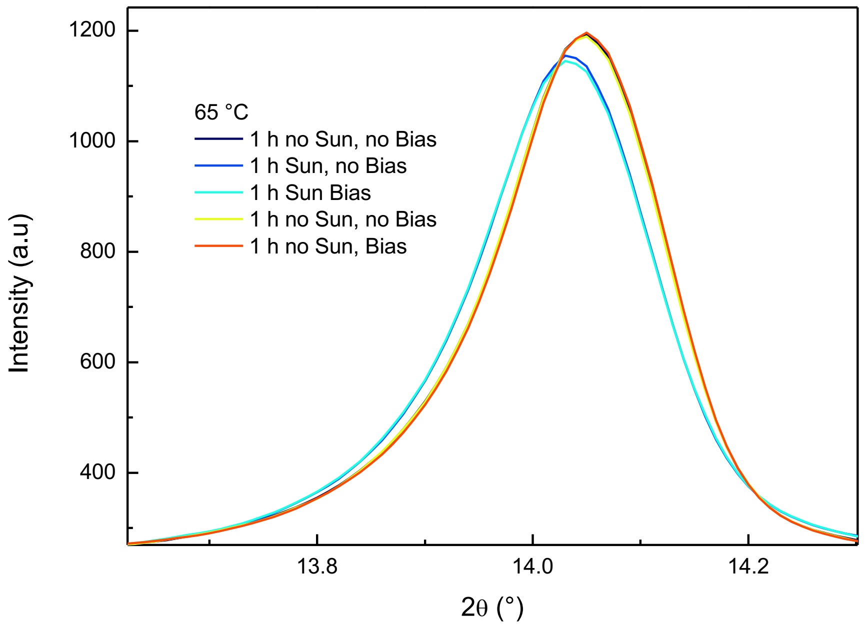

Figure SI8: shift of the XRD peak under illumination and bias at MPP. At a constant temperature of 65°C, a peak shift occurs immediately after one hour of illumination without bias. Following an additional hour of exposure to sunlight and MPPT, no significant changes are found. After another hour without sunlight and bias, the peak returns to its initial position and intensity. An additional hour of bias applied at MPP in the dark results in no peak shift

Supplementary note 2

We note that in Ref 5, intensity and FWHM behave like in our case if considering an initial time interval of 1h. In that paper, the authors tend to exclude ionic migration along the timescale of <3h in favour of less distorted Pb-I-Pb bonds or elongation of Pb-I bonds which causes lattice expansion. They also comment that light-induced lattice expansion is the dominant mechanism for lowering the interface barrier and improving the V_{oc} and FF. We instead prolonged the experiments to 20h and we found a progressive lowering (from t>1h) of the V_{oc} and an increase in J_{sc} both consistent (but not only related) with the PL data (bandgap shrinkage[6]). Tsai et al[1] similarly observed a redshift of the PL peak of 5meV in a time interval <3h, compared to 47meV found after 20h in our case. In both cases, a restore of the lattice parameters after switching off the sunlight is observed. Considering differences and analogies, we agree that a possible interpretation of the phenomenon is the weakening of the Pb-I bonds with consequent local movements of ions activated by light. Local ionic movement, interpreted as short/medium range (fully or partially) reversible migration of the fastest (or more mobile) ionic species, enables a unifying key to read for local demixing and change of composition depending on the involved species, intrinsic defects (arising from the preparation procedures), and eventually on the working temperature (stress tests applied hereafter). The demixing phenomenon is intrinsically time-dependent in the time scale from minutes to hours and can bring different scenarios in long-term device operation.

Supplementary References

1 Lamanna, E. et al. Mechanically Stacked, Two-Terminal Graphene-Based Perovskite/Silicon Tandem Solar Cell with Efficiency over 26%. *Joule* **2020**, *4*, 865-881
2 Cacovich, S. et al. Gold and iodine diffusion in large area perovskite solar cells under illumination. *Nanoscale*, **2017**, *9*, 4700-4706
[3] Reddy, S.H. et al. Holistic Approach toward a Damage-Less Sputtered Indium Tin Oxide Barrier Layer for High-Stability Inverted Perovskite Solar Cells and Modules. *ACS Appl. Mater. Interfaces* **2022**, *14*, 45, 51438–51448
4 Shlenskaya, N.N. et al. Light-induced reactivity of gold and hybrid perovskite as a new possible degradation mechanism in perovskite solar cells. *J. Mater. Chem. A,* **2018**, *6*, 1780-1786
5 Tsai, H. et al. Light-induced lattice expansion leads to high-efficiency perovskite solar cells. *Science* **2018**, *360*, 67–70
6 Jacobsson, T.J.; Hultqvist, A.; García-Fernández, A. et al. An open-access database and analysis tool for perovskite solar cells based on the FAIR data principles. *Nat. Energy* **2022**, 7, 107–115 https://doi.org/10.1038/s41560-021-00941-3
